# The Utility and Limitations of Contrast-Enhanced Ultrasound for the Diagnosis and Treatment of Prostate Cancer

**DOI:** 10.3390/s150304947

**Published:** 2015-02-27

**Authors:** Futoshi Sano, Hiroji Uemura

**Affiliations:** Department of Urology, Yokohama City University Graduate School of Medicine, 3–9, Fukuura, Kanazawa-ku, Yokohama 236-0004, Japan; E-Mail: snfts7974@gmail.com

**Keywords:** prostate cancer, contrast-enhanced ultrasound, targeted biopsy, microbubbles

## Abstract

In association with the widespread use of prostate specific antigen (PSA) screening, the numbers of men identified with early-stage prostate cancer (PCa) are increasing in the developed countries, including Japan. However, the accurate localization of PCa lesions in diagnostic imaging is still difficult because PCa has a tendency to be multifocal in the prostate gland. Contrast-enhanced ultrasound (CEUS) improves the detection of PCa by visualizing cancerous lesions in order to target a needle biopsy. CEUS has the potential to enable not only accurate diagnoses but also novel treatments such as focal therapy. The combination of CEUS and other modalities is expected to improve the diagnosis of PCa and its treatment.

## 1. Introduction

Prostate cancer (PCa) is the most common solid neoplasm among males in the United States and in the countries of the European Union [1]. In the diagnosis of PCa, grayscale transrectal ultrasound (TRUS) has generally been used for prostate biopsies. Ultrasound is superior to other imaging modalities in accessibility, noninvasiveness and cost. TRUS can make better images of the prostate than transabdominal ultrasound. Thus, TRUS is essential in the diagnosis and treatment of PCa. However, it is difficult to detect PCa by using standard grayscale or Doppler imaging TRUS because PCa lesions cannot be isolated with sufficient accuracy [2]. Therefore, a systematic multisite biopsy guided by TRUS is the standard procedure for biopsy of the prostate gland. For the initial diagnosis, a core biopsy of 10–12 systematic transrectal or transperineal peripheral zone biopsies should be performed under ultrasound imaging guidance [1]. However, this method is not adequately effective because the biopsies are sampled without the identification of cancer-suspicious lesions, and the risk of overlooking pathological tissue cannot be ignored [3].

## 2. Contrast-Enhanced Ultrasound (CEUS)

To improve the results of biopsies using US, contrast harmonic imaging has been developed [4,5]. PCa tissue is associated with increased microvessel density due to its proliferation of neovessels [6,7]. If these changes in tissue blood flow could be visualized, the accuracy for detecting PCa could potentially increase. Microbubble US contrast agents are administered intravenously, pass through the pulmonary circulation and then enhance vascular end organs. The second-generation US contrast agents consist of small encapsulated gas bubbles which can be used at a lower mechanical index and have longer enhancement durations. Contrast-specific imaging techniques enable differentiation between the non-linear signals reflected by the microbubbles and the linear signals from the tissue. The addition of microbubbles as additional reflectors into the bloodstream increases the sensitivity of imaging. These techniques are capable of detecting single microbubbles and can therefore visualize the blood flow in the microvasculature. The first application of CEUS was in the heart for myocardial perfusion measurement [8]. In addition, CEUS is now used mainly for the detection of liver malignancies.

**Figure 1:**
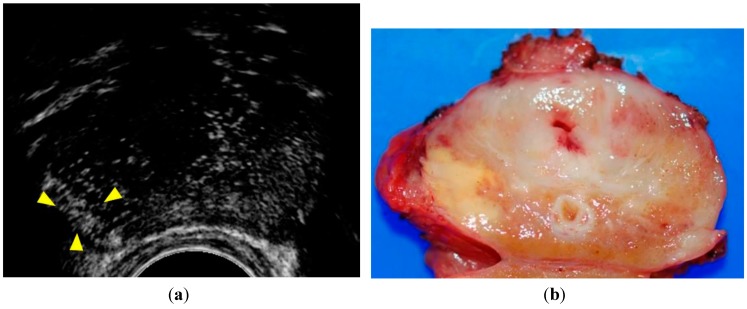
Patient with a PSA level of 16.2 ng/mL. (**a**) CEUS showed rapid contrast enhancement in the peripheral zone of the right lobe (arrowheads); (**b**) Surgical specimen of the case shown in this case. A yellow tumor was shown in the right lobe in agreement with the enhancement of the lesion. Pathological examination revealed that the tumor was a Gleason 3 + 4 adenocarcinoma.

In previous reports, CEUS findings suggestive of cancer have been defined as rapid contrast enhancement, increased contrast enhancement, and asymmetric intraprostatic vessels [9,10] (Figure 1).

**Figure 2:**
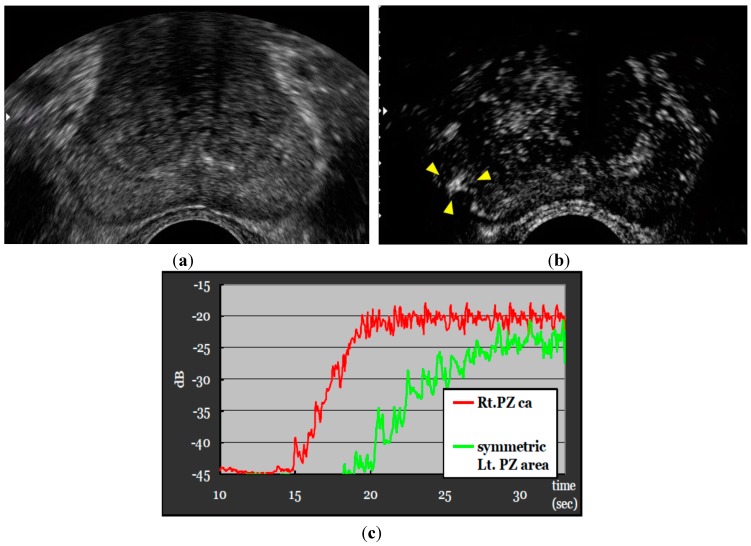
Patient with a PSA level of 7.4 ng/mL. Strong and rapid contrast enhancement was noted in the PZ of the right lobe. Biopsies of the corresponding region resulted in Gleason score of 3 + 3. The time intensity curve showed the early contrasting effect of the cancerous lesion in comparison with the symmetric normal tissue of the left lobe. Gleason score of 4 + 3. (**a**) Gray scale; (**b**) CEUS; (**c**) time intensity curve.

**Figure 3:**
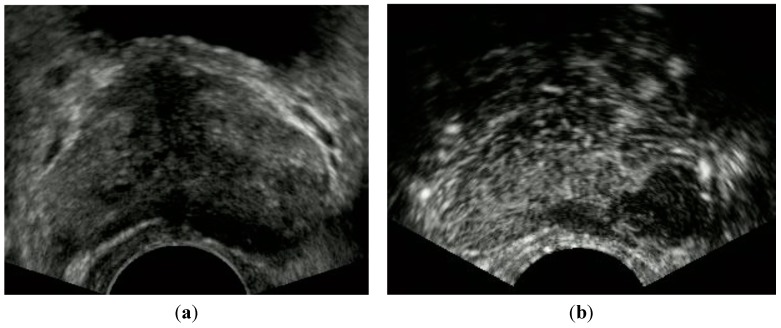
Patient with a PSA level of 13.8 ng/mL. CEUS showed an unenhanced nodule in the PZ of the left lobe. Biopsies of the corresponding lesion resulted in adenocarcinoma with Gleason score of 4 + 3. (**a**) Gray scale, (**b**) CEUS.

Figure 2 shows the time intensity curve, resulting in the early contrasting effect of the cancerous lesion in comparison with the symmetric normal tissue of the left lobe. However, according to other studies, some tumors could also be visualized as no or low enhancement compared to the surrounding tissue [11,12]. Figure 3 indicates CEUS showing an unenhanced nodule in the PZ of the left lobe. When a targeted biopsy is performed, it seems advisable to inspect the areas of no or low enhancement.

The pathologic evaluation of radical prostatectomy specimens is essential for cancer foci detection (Table 1). Matsumoto *et al.* compared radical prostatectomy specimens with pre-operatively performed grayscale US, power Doppler and harmonic imaging CEUS. In the 50 prostate glands studied, a total of 104 PCa foci were found at pathologic evaluation. Grayscale imaging demonstrated 21 (20.2%) and CEUS imaging demonstrated 32 (30.8%) of these foci [13]. Strazdina *et al.* conducted a similar study in 50 patients who were scheduled to undergo radical prostatectomy. Grayscale US demonstrated 34 of the 72 cancer foci (47.2%), and CEUS demonstrated 44 foci (61.1%) [14]. These results indicate that CEUS provides improved PCa detection compared to conventional grayscale. However, the sensitivity of CEUS is lower in cases of small low-grade tumors and centrally located lesions.

**Table 1:** CEUS studies of prostate cancer (whole prostate specimens).

Authors	No. of Patients	PSA Level (ng/mL)	Number of Foci Revealed			Peripheral Zone		Transition Zone	
			Pathology	Grayscale	CEUS	Sensitivity	PPV	Sensitivity	PPV
Strazdina *et al*. [14]	50	9.31 (±4.96)	72	34 (47.2%)	44 (66.1%)	72.2% *	86.7% *	27.8%	N/A
Matsumoto *et al*. [13]	50	7.8 (2.8–24.8)	104	21 (20.2%)	32 (30.8%)	42.9%	N/A	12.2%	N/A

* Peripheral zone or invaded both the peripheral and central gland; N/A: no assessment.

## 3. Targeted Biopsy Using CEUS

Table 2 shows the results of recent studies of targeted biopsy [12,15,16]. Xie *et al.* examined the cancer detection rate of grayscale, power Doppler or CEUS. For the per-patient comparison, CEUS alone did not significantly improve the overall performance over that of grayscale, power Doppler. However, the combination of these three examinations could detect more patients with PCa compared with grayscale, power Doppler [14]. Our previous study showed that CEUS increased the detection rate of PCa in a multicenter clinical trial, indicating that diagnosis using targeted biopsy together with systematic biopsy will be useful for PCa detection [15]. Zhao *et al.* demonstrated the potential for improvement of cancer detection rate by CEUS, and concluded that systematic biopsies should not be eliminated [16].

One of the disadvantages of CEUS is the subjective interpretation by the investigator. In most of the previous studies, the diagnostic criteria of a cancerous lesion, such as early enhancement, relied on the visual impression of the examiner. Reproducibility of the examination results is very important. Therefore, not only subjective but also quantitative evaluations are required. Jiang *et al.* [17] examined factors that influenced the degree of enhancement of PCa on CEUS, and they found that the development of neovascularity in PCa was demonstrated by an increased peak intensity on CEUS imaging. Jiang *et al.* showed that the peak intensity of tumor foci was significantly higher than that of benign prostatic hyperplasia (BPH) lesions. Their study also revealed that the location and Gleason score of tumor foci were the influencing factors of the peak intensity value [17].

**Table 2:** CEUS studies of prostate cancer (prostate biopsy specimens).

Authors	No. of Patients	PSA Level (ng/mL)	Systematic Bx	Targeted Bx (CEUS)	Systematic Bx + Targeted Bx
Xie *et al*. [12]	150	22.09 (4.16–85.8)	57 (38.0%)	63 (42.0%)	73 (48.7%)
Uemura *et al*. [15]	71	8.77 (4.37–19.9)	22 (31.0%)	23 (32.4%)	30 (42.3%)
Zhao *et al*. [16]	65	10.7 (0.5–100)	27 (41.5%)	23 (35.4%)	29 (44.6%)

Bx: biopsy.

Jung *et al.* [18] evaluated PCa lesions using CEUS in a perfusion analysis. Twenty patients with biopsy-proven PCa underwent CEUS prior to a radical prostatectomy. Based on perfusion-related parameters (mean transit time, rise time, and wash-in rate), color-coded parametric images were made in order to define cancerous lesions. Areas of abnormal values can be shown as hot spots in such images. A pathological examination revealed 34 PCa foci in the 20 patients. In 30 of the 34 foci, an early enhancement within the tumor was detected by evaluating parametric images [18]. In conclusion, it is possible that a combination of visual impression and quantitative assessment leads to the improvement of the performance of targeted biopsies. Currently, perfusion analysis is limited to one cross-section because the probe is two-dimensional (2D). In the future, with the use of a 3D probe, the accuracy of perfusion analysis may be further improved.

## 4. Tumor Size and Pathology Related to CEUS Imaging

As mentioned above, CEUS has become a very useful diagnostic tool for PCa detection, but a number of limitations remain. As Strazdina *et al.* reported, CEUS detected only 35.5% of low-grade and 80% of intermediate-grade PCa, while the corresponding results of gray-scale imaging were 16% and 70%, respectively. They concluded that the sensitivity of CEUS was lower in cases of small low-grade PCa [14]. Although Aigner *et al.* reported high diagnostic sensitivity (100%) and negative predictive value (99.8%) for CEUS [10], the tumor size of those diagnosed lesions was medium (approx. 1–3 cm), especially in the peripheral zone. Moreover, the transition zone tumor is difficult to identify by CEUS because hypervascular lesions of BPH occupy the transition zone. Of course, the larger malignant tumors were more easily detected by CEUS [9].

Contrast-enhanced findings of large tumors by CEUS showed various patterns according to the difference of tumor vascularity. Along with the increase of tumor size, the total blood volume is occasionally decreased [19]. It should thus be noted that various patterns of contrast-enhanced findings in PCa are shown by CEUS, as we previously reported [11,15].

The correlation between the pathological malignancy, *i.e.*, the Gleason score, and CEUS findings has been examined. One report indicated that lower malignant tumors (with <1 cm) were less visible and that the average Gleason score for PCa foci which were detected by CEUS was 5.1, whereas that for foci undetected by CEUS was 4.4. In addition, those authors suggested that the majority of tumors detected by CEUS had larger-sized tumor and higher Gleason scores than those undetected by CEUS, and similar results have been reported in other investigations [20,21]. These results are in agreement with a recent study that analyzed prostatectomy specimens and found that larger-sized tumors in the transition zone showed higher Gleason scores in Japanese patients compared to patients in the USA [22].

## 5. Limitations of CEUS Imaging for Targeted Biopsies

Although CEUS seems a promising technique in the diagnosis of PCa, it still has some disadvantages to be addressed. A review of the detection rate of targeted biopsies for PCa revealed that targeted biopsy by CEUS alone provides no significant increase in the detection rate compared to systematic biopsy, and that the detection rate of CEUS is better when combined with systematic and targeted biopsies compared to systematic biopsy alone [23]. Vourganti *et al.* reported that like CEUS, MRI/ultrasound fusion targeting biopsy resulted in pathological upgrading by 38.9% compared with standard trans-rectal ultrasound biopsy [24].

Currently, the superiority of targeted biopsy by modalities such as CEUS and MRI is seemingly limited in the case of repeat biopsy, according to some studies [24,25,26]; in particular for MRI. An important thing regarding the detection of PCa is to avoid overdetection and overtreatment. This requires the ideal diagnostic procedure that not only identifies significant PCa lesions but leaves no significant (indolent) cancer lesions undetected.

## 6. The Usefulness of CEUS for the Treatment of PCa

Apart from its use as a diagnostic tool before prostate biopsy, CEUS has been considered as a distinctive procedure to assist the provision of treatments such as brachytherapy. In general, the implantation of seeds has been performed in whole prostate gland in accord with the concept that cancer lesions are usually multifocal and that insignificant/small lesions are difficult to identify using the standard radiographic procedures. A recent report suggested that CEUS imaging can be used to support brachytherapy planning [27]. US techniques and quantitative imaging are more advanced than ever [9,28] and its high accuracy for PCa detection confirms the utility of CEUS for PCa radiotherapy [29,30]. By using CEUS for brachytherapy planning, Pieter *et al.* demonstrated the usefulness of CEUS to cover better lesion without any increase in the radiation dose [27]. Thus the identification of intraprostatic lesions by CEUS provides improved coverage of intraprostatic lesions without increasing the radiation dose.

In accord with its increased sensitivity for detecting PCa, CEUS can play an important part in the follow-up modality after each treatment. It is well known that gas-encapsulated microbubbles with a dia. <10 µm can penetrate the microvascular system and act as intravascular reflectors. In general, CEUS is able to visualize the hemodynamic properties in where are rich in angiogenesis, increased vascularity and abnormal blood flow patterns particular in such as cancer tissue [31,32]. Additionally, CEUS can be used as the follow-up monitoring tool after a treatment to examine the treated area, which usually shows the absence of blood signals.

Wondergem demonstrated that CEUS imaging showed a lower region of the prostate corresponding to the area treated with high-intensity focused ultrasound (HIFU) [33]. In addition to HIFU, cryosurgery and brachytherapy are appropriate for local therapy against a small-focus adenocarcinoma of the prostate gland, and in such cases CEUS seems a promising technique for the follow-up of prostate conditions and treatment. Another group proposed a similar use of CEUS to monitor the outcome of hormonal therapy, in a study revealing that the vascular enhancement of the carcinoma declined with therapy, as did the level of PSA [34]. However, further improvement of the sensitivity and specificity of PCa detection by CEUS is required to appropriately monitor the follow-up of PCa after treatment.

The microbubbles used in CEUS have the unique roles of both a contrast agent and also providing drug delivery assistance [35]. This new application is known as sonoporation, which is not completely understood, but being investigated as a novel approach for drug or gene delivery. Ultrasound may destroy microbubbles, resulting in the creation of microjets that open a pathway through the cell membrane and increase cell permeability. Gas microbubbles also strengthen gene transfer by increasing cavitation. Sonoporation by microbubbles is thus expected to provide an efficient direct transfer of drugs or genetic materials into the cytoplasm.

Mehier-Humbert *et al.* reported that fluorescent nanospheres could enter cells when sonoporation was applied [36]. Interestingly, another investigation demonstrated the uptake of an antisense oligonucleotide in prostatic cancer xenografts in nude mice after the intravenous injection of loaded microbubbles which were subsequently exposed to ultrasound [37]. The application of microbubbles in both diagnosis and therapy is expected to develop further and become acceptable as a routine tool.

## 7. The Potential Use of CEUS for the Diagnosis of Genitourinary Malignancies

Regarding the detection of bladder cancer, CEUS was reported to be significantly more accurate than B-mode ultrasound, but although the sensitivity of CEUS was likely to be better than that of baseline ultrasound per number of tumors (88.37% *vs*. 72.09%), for tumors less than 5 mm in size its sensitivity was dramatically very low (20%) [38]. A similar limitation of ultrasound was reported; *i.e.*, the detection of bladder lesions <1 cm by three-dimensional ultrasound showed low sensitivity [39]. Similar to the location-dependent difficulty of PCa detection, e.g., in the transition zone, the detection of small tumors in the bladder neck (floor) is thought to be difficult in males, particularly those with a large prostatic central gland protruding into the bladder lumen [40]. We also observed intense enhancement in the transition zone in the prostate by CEUS imaging, due to the zone’s arterial neovascularization [41]. In brief, CEUS can significantly clarify the existence of bladder cancer to the extent of a certain tumor size compared with baseline ultrasound, especially in uncertain baseline ultrasound cases.

Compared with bladder cancer, CEUS imaging of urothelial carcinoma in the renal pelvis is likely to be characteristic due to the poor blood supply. Such imaging includes slow-in, fast-washout and hypo-enhancement properties, which makes the diagnosis more efficient [42]. Renal cell carcinoma (RCC) with its rich blood supply has often shown synchronous-in, hyper- or iso-enhancement at peak enhancement, and heterogeneous enhancement [43]. Enhancement patterns are depicted differently by CEUS depending on the tumor size; e.g., a heterogeneous pattern is shown mainly in tumors >3 cm [43,44]. The benign renal tumor angiomyolipoma shows a homogeneous pattern by CEUS, suggesting that CEUS may have the capability to differentiate benign from malignant tumors that are >3 cm.

Radiofrequency (RFA) and cryoablation (CA) are now used widely with the aim of preservation of renal function; however, these procedures hold the risk of residual or recurrent tumors [45]. A prospective study by Barwari *et al.* indicated that CEUS in combination with contrast pulse sequencing could accurately reveal the absence of an enhancing lesion in an ablated renal mass [46]. CEUS may thus be useful to monitor tumor recurrence.

## 8. Conclusions

Although CEUS improves the sensitivity of PCa detection, the targeted biopsy alone with CEUS imaging cannot yield a higher detection rate of PCa. However, a satisfactory detection rate seems to be obtained when systematic and targeted biopsies are combined. At present, CEUS is reasonable for patients with elevated PSA levels and a previous negative biopsy. If this technique of CEUS can be developed further, it will contribute to the monitoring of PCa after treatment and to the accurate identification of cancer lesions when focal therapies might be performed. As a promising application of CEUS, microbubbles of CEUS have the potential to play a role in the delivery of drugs or genes locally, an application that must be further investigated. The uses of CEUS for both diagnosis and therapy will create a new era of cancer treatment, especially for PCa.
